# Improved Survival Outcome and Access to Cancer Screening from Hemorrhoid in Patients with Rectal Cancer

**DOI:** 10.1155/2020/5045142

**Published:** 2020-12-10

**Authors:** Qi Zou, Donglin Ren, Xiaolin Wang, Liangliang Bai, Guannan Tang, Meijin Huang, Yanxin Luo, Huichuan Yu

**Affiliations:** ^1^Guangdong Institute of Gastroenterology, Guangdong Provincial Key Laboratory of Colorectal and Pelvic Floor Disease, The Sixth Affiliated Hospital, Sun Yat-sen University, Guangzhou, Guangdong 510655, China; ^2^Department of Colorectal and Anal Surgery, The Sixth Affiliated Hospital, Sun Yat-sen University, Guangzhou, Guangdong 510655, China; ^3^Department of Colorectal Surgery, The Sixth Affiliated Hospital, Sun Yat-sen University, Guangzhou, Guangdong 510655, China

## Abstract

**Background:**

The interventions for hemorrhoid increase access to rectal cancer screening and thus might reduce cancer death. We aimed to examine the impact of hemorrhoid on survival outcomes in rectal cancer.

**Methods:**

We identified 510 patients with stage I to III rectal cancer from a prospectively collected database. Patients were divided into hemorrhoid and non-hemorrhoid group. The primary endpoints were disease-free survival (DFS) and overall survival (OS).

**Results:**

Hemorrhoid group had significantly more stage I-II diseases in comparison to nonhemorrhoid group (71.1% vs. 55.9%, *P* = 0.049). The hemorrhoid group had significantly better DFS and OS compared to nonhemorrhoid group, the hazard ratios (HRs) of which were 0.39 (95% CI 0.17-0.88, *P* = 0.018) and 0.33 (95% CI 0.12-0.92, *P* = 0.034), respectively. Multivariate analysis revealed that hemorrhoid was independently associated with DFS [adjusted HR 0.43 (95% CI 0.17-0.95, *P* = 0.045)]. A nomogram for predicting DFS outcome was generated based on hemorrhoid history, with a concordance index of 0.71 (95% CI 0.66-0.75, *P* < 0.001).

**Conclusions:**

There may exist a screening effect and survival benefit from hemorrhoid in rectal cancer, which supports the significance of rectal cancer screening in lowering its mortality.

## 1. Introduction

Colorectal cancer (CRC) is one of the major causes of cancer death [1], with estimated 881,000 deaths in 2018 [2]. Current screening, including colonoscopy, sigmoidoscopy, and fecal testing [3], has dramatically reduced CRC incidence and mortality by detection of precancerous diseases and early-stage CRC over the past several decades [1, 4]. Unfortunately, there remain large amounts of average-risk people having not been screened even in developed countries [5]. The previous studies have revealed screening failures for CRC are common in populations, including never screen, failure to screen at appropriate intervals, and failure to receive surveillance [6, 7]. The estimated 46%-63% CRC deaths occur when patients fail to screen [8]. This condition can be attributed to multiple barriers, including lack of physician referral, financial barriers, and patients' adherence and examining fears [9–12].

Hemorrhoid is one of the most prevalent diseases and a leading cause of ambulatory care visits worldwide [13–15]. Rectal cancer and hemorrhoids shared several signs or symptoms, including bleeding from the rectum, blood mixed with stool, and a change in bowel habits [16]. As a result, if one patient had some of these shared complaints, a digital examination along with colonoscopy or other CRC screen strategies is recommended, even for diagnosed hemorrhoid, in clinical practice. Hemorrhoid has been recognized as one of the most common factors associated with negative colonoscopy result in CRC screen [17]. We therefore proposed that patients suffered from hemorrhoid may get benefit from screening and thus have CRC found at an earlier stage and have less CRC death. Herein, we conducted a retrospective cohort study to examine the impact of symptomatic hemorrhoid on long-term survival outcomes in rectal cancer.

## 2. Materials and Methods

### 2.1. Patients and Cohort

We retrospectively identified and included rectal cancer patients with stage I to III who underwent curative resection from the prospectively and consecutively collected database in our institution from June 2007 and June 2012. The data from inpatient medical records were evaluated, including the presence of symptomatic hemorrhoid history, demographic characteristics, potential prognostic variables, surgical procedures, and regimens. The patients with multiple primary cancers, familial adenomatous polyposis (FAP), inflammatory bowel disease (IBD), and failed evaluation of hemorrhoid were excluded.

### 2.2. Preoperative Evaluation

All the patients in this cohort were prospectively evaluated by a surgeon trained in colorectal and anal specialty according to the protocol of the institutional database program of colorectal disease as previously applied [18, 19]. In brief, the symptoms and signs, endoscopy, and disease history of each patient were evaluated and recorded. Based on these evaluations, patients in our study were divided into hemorrhoid and nonhemorrhoid group. Patients were classified into hemorrhoid group if they met any one of the following criteria: (1) patient-report diagnosis of symptomatic hemorrhoids in previously outpatient care or hospitalization, (2) colonoscopy-report diagnosis of hemorrhoids with symptoms, and (3) surgeon-report prolapse or bleeding of hemorrhoids in physical examination. Patients meeting none of the above criteria were classified into nonhemorrhoid group. According to the report from endoscopy and anorectal surgeons, hemorrhoids can be diagnosed as internal, external, or mixed. In this cohort, patients received colonoscopy if they had any of hematochezia, positive fecal occult blood tests (FOBT), alteration of bowel habits, unexplained abdominal pain, old age (>50 years), and suspected colorectal disease (e.g., tumor or IBD).

The stages of rectal cancer were classified according to the American Joint Committee on Cancer (AJCC) staging [20]. Staging procedures, including contrast-enhanced computed tomography scans of the thorax, abdomen, and pelvis; pelvic MRIs; and colonoscopy, were performed in all cases to exclude patients with evidence of distant metastatic disease at the initial diagnosis. Moreover, we used neutrophil-to-lymphocyte ratio [21] and platelet-to-lymphocyte ratio [22] to evaluate patients' preoperative systemic inflammation status.

### 2.3. Follow-Up and Study Endpoints

Patients were treated and followed up according to NCCN guidelines-based protocols in our institute [23]. The primary endpoints were disease-free survival (DFS) and overall survival (OS). DFS was defined as the time from the surgery to local recurrence, distant metastasis, or death from any cause. OS was defined as the time from the surgery to death. Total recurrence was defined as patients with local recurrence and/or distant metastasis during follow-up after operation. The secondary endpoints included anastomotic complication, postoperative fever, and pelvic abscess. Anastomotic complication refers to anastomotic leakage, bleeding, or stenosis after operation in this study. Postoperative fever was defined as a body temperature ≥38.5°C within seven days after operation.

### 2.4. Statistical Analyses

Age and body mass index were analyzed as continuous variables, and other factors were analyzed as categorized variables. The intergroup comparisons of variables were conducted using the analysis of variance or Kruskal–Wallis tests for continuous variables according to their distributions, and the chi-square and two-tailed Fisher's exact tests for categorized variables. The Kaplan-Meier analysis was applied to estimate long-term survival after curative surgery. A univariate screen of prognostic association with DFS or OS by Cox proportional hazard model for each variable extracted from medical records was applied, and the prognostic factors were included in the multivariate Cox model to examine their independent associations with DFS or OS. Based on this model, a nomogram subjected to internal validation set was generated for predicting 3-year and 5-year DFS, and the concordance index (C-index) was calculated to evaluate the predictive accuracy. Data analyses were performed using the R software 3.6.1. *P* values < 0.05 were considered statistically significant with two-tailed tests.

## 3. Ethics Statement

This study was approved by the institutional Medicine Ethics Committee. Informed consents were obtained from all human subjects in accordance with The World Medical Association Declaration of Helsinki.

## 4. Results

### 4.1. Baseline Clinicopathologic Features

**Figure 1:**
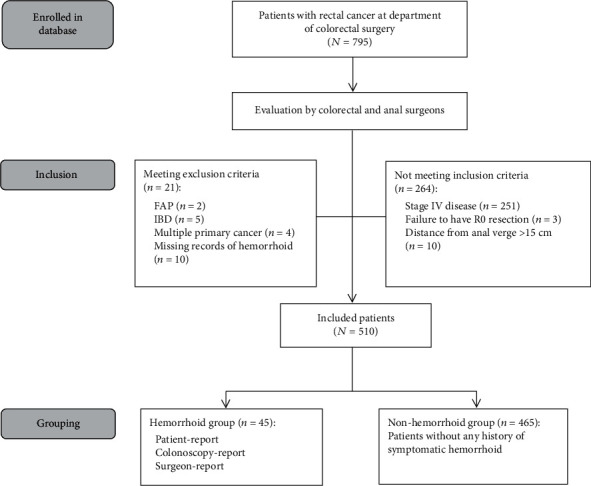
Flow diagram of patients disposition in the association analysis between hemorrhoid history and outcomes in rectal cancer.

Between June 2007 and June 2011, 795 patients with rectal cancer were recognized, and their data were collected from the electronic database and medical records. Among them, a total of 510 patients matched the inclusion and exclusion criteria and were included in this study (Figure 1). The baseline characteristics were shown in Table 1. There were 295 (57.8%) male and 215 (42.2%) female patients, with the median age of 59 years (range, 21 to 89 years). The AJCC staging among the patients was distributed as 57.3% and 42.7% for stages I-II and III, respectively.

### 4.2. Symptomatic Hemorrhoid and Tumor Features

There were 45 (8.8%) patients in the hemorrhoid group. Among them, 31 (68.9%) cases were patient-report diagnosis of symptomatic hemorrhoids in previous care, and 14 (31.1%) cases were grouped based on endoscopy- or surgeon-report. Overall, the demographic, patient-reported symptoms, baseline systemic inflammation status, therapeutic, and clinicopathological characteristics were similar between two groups, except for the TNM stage (Table 1). Expectedly, the early-stage (stage I-II) disease had a significantly higher rate (71.1% vs. 55.9%, *P* = 0.049) in hemorrhoid group when compared with nonhemorrhoid group. Of note, hemorrhoid group had 11.9% lower rate of T3-4 stage disease, although the difference was not significant (*P* = 0.100). For tumor location, hemorrhoid group had more tumors in lateral rectal wall (15.6% vs. 8.2%, *P* = 0.101) and fewer tumors in posterior rectal wall (6.7% vs. 17.8%, *P* = 0.056), although these differences were not significant.

### 4.3. Short-Term and Long-Term Outcomes in Patients with and without Symptomatic Hemorrhoid

We next investigated the potential impact of symptomatic hemorrhoid on surgical complications after resection for rectal cancer. The incidence of anastomotic leakage and total anastomotic complication was 6.9% (35/510) and 13.1% (67/510), respectively, and they were similar between hemorrhoid and nonhemorrhoid group. For infective complications, the incidence of postoperative fever and pelvic abscess was 30.4% (155/510) and 3.9% (20/510) in the whole cohort, respectively, and they were similar between two groups (Table 2).

In the long-term outcomes, the incidence of total recurrence, local recurrence, and distant metastasis was 27.8% (142/510), 10.7% (53/495), and 20.2% (100/495), respectively. Patients in hemorrhoid group had 15.9% lower incidence of total recurrence compared with nonhemorrhoid group [13.3% (6/45) vs. 29.2% (136/465), *P* = 0.023] (Table 2). Of note, significantly lower distant metastasis rate [8.9% (4/45) vs. 21.3% (96/450), *P* = 0.047] was observed in hemorrhoid group, while the local recurrence rate [8.9% (4/85) vs. 10.9% (49/450), *P* = 0.150] was similar in two groups (Table 2).

### 4.4. Univariate Cox Survival Analysis for Symptomatic Hemorrhoid and Other Prognostic Factors

**Figure 2:**
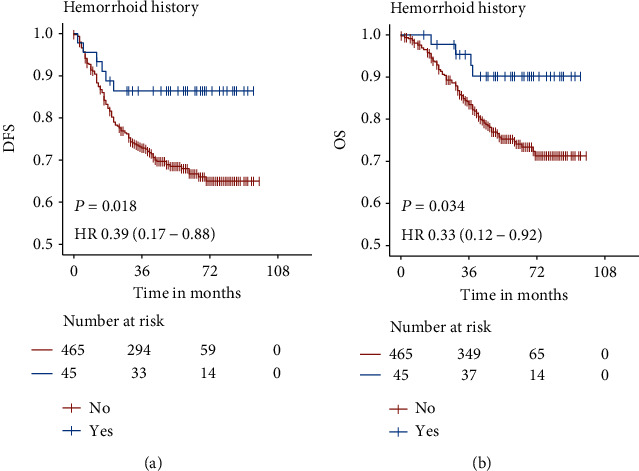
Kaplan–Meier survival curves of rectal cancer patients with or without hemorrhoid history. The curves showed significantly higher DFS (a) and OS (b) outcomes in the hemorrhoid group compared to the nonhemorrhoid group. Log-rank test *P* values and hazard ratios (HRs) with 95% CI are given in each plot. DFS: disease-free survival; OS: overall survival.

Kaplan–Meier curves showed significant higher 3-year DFS (86.4% vs. 72.8%, *P* = 0.018) and OS (95.3% vs. 83.5%, *P* = 0.034) rate in hemorrhoid group in comparison to nonhemorrhoid group (Table 3, Figure 2). The Cox univariate analysis indicated that elder age, advanced AJCC stages, low-grade differentiation, lymphovascular invasion, perineural invasion, elevated CEA, and symptomatic hemorrhoid were significantly associated with increased mortality (Table 3). Specifically, hemorrhoid group had significantly better DFS and OS outcomes compared to nonhemorrhoid group, the hazard ratios (HRs) of which were 0.39 (95% CI 0.17-0.88, *P* = 0.018) and 0.33 (95% CI 0.12-0.92, *P* = 0.034).

### 4.5. Multivariate Cox Survival and Sensitivity Analysis

**Figure 3:**
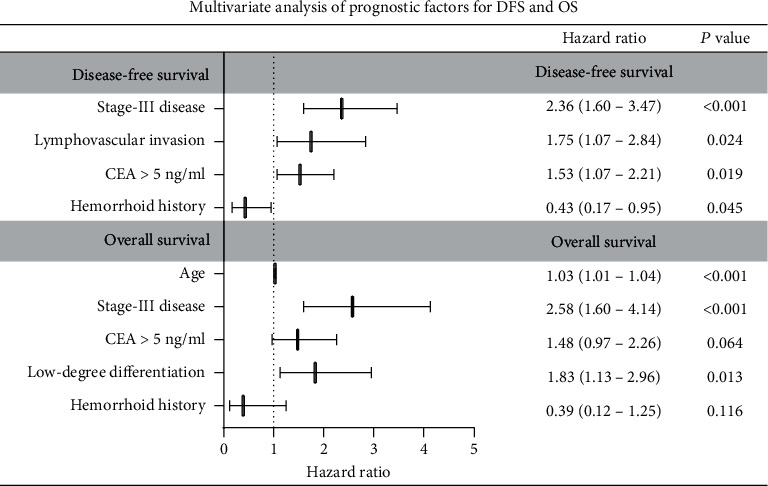
Multivariate analysis of prognostic factors for DFS and OS. The forest plot for the hazard ratios and 95% confidence intervals of each predictor in the multivariate Cox model for DFS and OS. CEA: carcinoembryonic antigen. HR: hazard ratio; CI: confidential interval.

Using the Cox multivariate analysis that included all significant variables in the univariate analysis, advanced stage, lymphovascular invasion, elevated CEA, and symptomatic hemorrhoid [adjusted HR 0.43 (95% CI 0.17-0.95, *P* = 0.045)] were still significantly associated with DFS outcome. Elder age, advanced stage, and low-degree differentiation had independently significant prediction value on OS. Although symptomatic hemorrhoid was still related to better OS outcome, it did not remain significant in this model [adjusted HR 0.39 (95% CI 0.12-1.25, *P* = 0.116)] (Figure 3).

**Figure 4:**
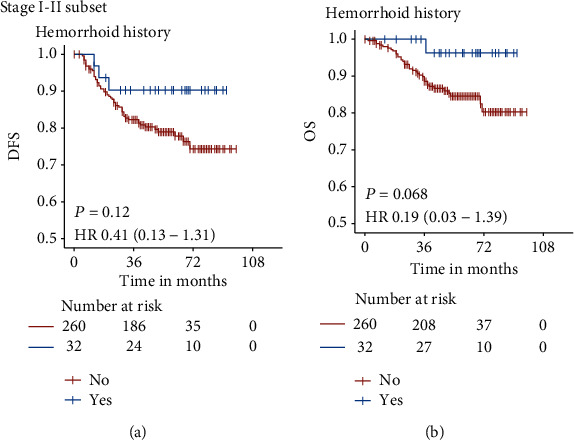
The association of hemorrhoid and survival outcomes in the early-stage subset. The Kaplan-Meier survival curves showed hemorrhoid group still had better DFS and OS outcomes in stage I-II subsets, although the Log-rank test was not significant. DFS: disease-free survival; OS: overall survival.

To determine if these better survival outcomes in hemorrhoid group depend on the higher rate of early-stage disease (stage I-II) attributed from the early-detection effect of hemorrhoid, further sensitivity analyses excluding stage-III disease were conducted. In the Kaplan-Meier analysis of a subgroup of 292 patients with early-stage disease, hemorrhoid group (32/292, 10.9%) still had a trend of better DFS and OS outcomes, although the results were not statistically significant (Figure 4). The adjusted HRs of DFS and OS were 0.41 (95% CI 0.13-1.31, *P* = 0.120)] and 0.19 (95% CI 0.03-1.39, *P* = 0.068), respectively, in the Cox regression model.

### 4.6. A Nomogram for Predicting Disease-Free Survival in Rectal Cancer

**Figure 5:**
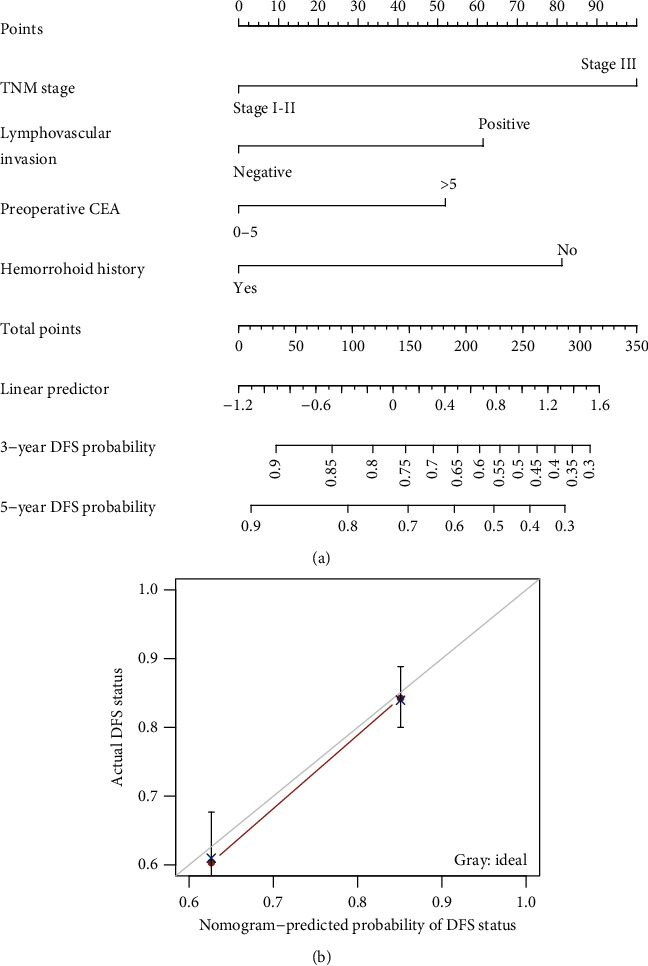
A nomogram and calibration curve for prediction of disease-free survival in rectal cancer. (a) A nomogram to predict individual patient-level 3-year and 5-year DFS based on hemorrhoid history and other clinicopathological risk factors. (b) Calibration plots for the internal validation of the nomogram. The observed DFS estimated by Kaplan-Meier was plotted against nomogram-predicted probability of DFS. 95% confidence intervals of the Kaplan-Meier estimates were indicated with vertical lines. Grayline indicated the reference line, showing where an ideal nomogram would lie. DFS: disease-free survival.

A nomogram for predicting 3-year and 5-year DFS outcome was generated using the independent variables in the multivariate Cox model, including symptomatic hemorrhoid, preoperative CEA, TNM stage, and lymphovascular invasion (Figure 5). The calibration curves for the nomogram were shown. The C-index of the nomogram for predicting DFS was 0.709 (95% CI 0.661-0.757, *P* < 0.001).

## 5. Discussion

To the best of our knowledge, this is the first study to directly demonstrate the association of symptomatic hemorrhoid with clinicopathological features and long-term survival outcomes in patients with rectal cancer. In consistent with our hypothesis, there exists a potential early-detection effect and survival benefit from hemorrhoid in rectal cancer after curative surgery. This survival benefit was still seen in early-stage CRC subset, and in multivariate analysis adjusted by TNM stage and other variables as well. Our results demonstrate the significance of screening in lowering CRC death. In addition, we found the favorable effect of symptomatic hemorrhoid on CRC was not only contributed by early detection; suggesting some pathophysiological changes in anorectal tissue of hemorrhoid patients may play a tumor-suppressor role in rectal cancer.

The interventions to differentially diagnose hemorrhoid from other diseases might increase access to screening, and the suffering from hemorrhoid might increase patients' compliance and adherence to screening uptake in care visits. Given that physician recommendation has been validated as one of the strongest predictors of CRC screening uptake [24], we are convinced that hemorrhoid might be the strongest predictor of screening uptake as well. A previous study revealed that failure to ever screen is the dominant type of screening failure, suggesting that improving uptake of screening remains important in decreasing CRC mortality [6]. In the present study, we found hemorrhoid group had higher early-stage rate and better survival outcomes, which supports this favorable effect of screening and suggests that interventions for hemorrhoid might reduce CRC death in populations.

The association between symptomatic hemorrhoid and better survival outcomes were independent of tumor stages and other variables, and the hemorrhoid group still had a trend of better OS and DFS results in the subset of early-stage rectal cancer. We therefore suppose that pathophysiological change in hemorrhoid patients may restrict the tumor progression. Several pathophysiological mechanisms of hemorrhoidal development have been postulated, including varicose veins in the anal canal and anal cushions disintegrate [25]. In addition, some physiological changes beyond anus-surrounding tissue have been observed. Patients with hemorrhoids had significantly lower rectal compliance, which may be attributed to the sustained contraction of bowel smooth muscle [26]. This physiological change may constrain the infiltration of cancer cells into deep bowel wall. The current surgical approaches, such as hemorrhoidectomy and stapled hemorrhoidopexy, can excise hemorrhoids and redundant mucosa and reverse pathophysiological changes of anal and lower rectal tissue [16]. However, the physiological change of proximal rectum may remain after these surgical approaches, and the protecting effect may be maintained. Our baseline characteristics comparison between groups support this interpretation, in which hemorrhoid group had 11.9% lower rate of tumor grown through the muscularis propria (T3-4 stage: 57.8% vs. 69.7%, *P* = 0.100).

The risk of CRC incidence in patients with hemorrhoid or other benign anal lesions has not been well documented. A retrospective cohort study revealed that the presence of hemorrhoids had an increased risk of developing CRC, with HR of 1.5 [27]. In contrast to this finding, we found a favorable effect of hemorrhoid on rectal cancer survival. This is not unexpected, since patients with hemorrhoid and individuals with high-risk of CRC may share several features, including obesity, lacking of movement, and low-fiber diet [28–30], and early-detection effect is the strongest protecting factor for CRC survival.

The strengths of our study include the comprehensive evaluation of all eligible CRC cases received by colorectal surgery specialty, the ability to accurately evaluate the current status and previous history of hemorrhoid for patients, and the use of manual review of patient medical records to construct hemorrhoid histories. However, we could not compare the exact data of screening rate in hemorrhoid patients and healthy individuals based on in-hospital data analysis, and further epidemiological investigations on screening interventions before admission to hospital are needed to confirm these findings. In addition, hemorrhoid is a prevalent disease in the general adult population, and it is supposed that some people with hemorrhoid do not complain about symptoms [14]. As a result, the patients in hemorrhoid group are “reported hemorrhoid” cases, which may weaken the finding that survival benefit could be independent of early-detection effect. However, the early-detection effect is provided by interventions from “reported hemorrhoid,” and the main finding of association among early detection, hemorrhoid, and survival benefits is still robust.

## 6. Conclusion

There may exist an early-detection effect and survival benefit from symptomatic hemorrhoid in rectal cancer, demonstrating the significance of screening in lowering CRC mortality. This finding needs to be further validated in epidemiological investigations on cancer screening before admission to hospital. In addition, we found this survival benefit from symptomatic hemorrhoid was not simply contributed by early detection, suggesting an underlying pathophysiological change in hemorrhoid patients may restrict tumor progression.

## Figures and Tables

**Table 1:** Distribution of baseline characteristics between patients with and without previous history of hemorrhoid.

Characteristic	Overall population	Previous history of hemorrhoid		*P* value
		No	Yes	
	(*N* = 510)	(*N* = 465)	(*N* = 45)	
Age-yr, median (range)	59 (21-89)	59 (21-89)	61 (33-77)	0.942
BMI, median (range)	22.1 (13.3-35.9)	22.1 (13.3-35.9)	22.4 (16.6-30.3)	0.971
Sex				0.533
Male	295 (57.8)	267 (57.4)	28 (62.2)	
Female	215 (42.2)	198 (42.6)	17 (37.8)	
Patient-reported symptoms				
Blood stools	352 (69.0)	318 (68.4)	34 (75.6)	0.321
Bowel habits change	110 (21.6)	101 (21.7)	9 (20.0)	0.789
Stools shape change	65 (12.7)	59 (12.7)	6 (13.3)	0.901
Abdominal pain	36 (7.1)	32 (6.9)	4 (8.9)	0.616
Time to symptom onset				0.964
0-6 months	348 (69.2)	317 (69.2)	31 (68.9)	
>6 months	155 (30.8)	141 (30.8)	14 (31.1)	
Tumor location: distance from anal verge				0.453
0-5 cm	207 (38.5)	192 (39.0)	15 (33.3)	
5-12 cm	330 (61.5)	300 (61.0)	30 (66.7)	
Tumor location: orientation				
Anterior wall	72 (14.1)	66 (14.2)	6 (13.3)	0.874
Posterior wall	86 (16.9)	83 (17.8)	3 (6.7)	0.056
Lateral wall	45 (8.8)	38 (8.2)	7 (15.6)	0.101
Circumferential	124 (24.3)	112 (24.1)	12 (26.7)	0.700
TNM stage, AJCC				0.049∗
I-II	292 (57.3)	260 (55.9)	32 (71.1)	
III	218 (42.7)	205 (44.1)	13 (28.9)	
T stage				0.100
T1-2	160 (31.4)	141 (30.3)	19 (42.2)	
T3-4	350 (68.6)	324 (69.7)	26 (57.8)	
Differentiation degree				0.192
Low	94 (19.1)	89 (19.8)	5 (11.6)	
Moderate/high	398 (80.9)	360 (80.2)	38 (88.4)	
Lymphovascular invasion				0.787
Negative	463 (90.8)	421 (90.5)	42 (93.3)	
Positive	47 (9.2)	44 (9.5)	3 (6.7)	
Perineural invasion				0.109
Negative	460 (90.2)	416 (89.5)	44 (97.8)	
Positive	50 (9.8)	49 (10.5)	1 (2.2)	
Preoperative CEA				0.849
0-5 ng/mL	327 (69.4)	298 (69.3)	29 (70.7)	
>5 ng/mL	144 (30.6)	132 (30.7)	12 (29.3)	
Preoperative neutrophil-to-lymphocyte ratio				0.620
<3	389 (78.9)	353 (78.6)	36 (81.8)	
>/=3	104 (21.1)	96 (21.4)	8 (18.2)	
Preoperative platelet-to-lymphocyte ratio				0.732
0-100	147 (29.5)	135 (29.7)	12 (27.3)	
>100	351 (70.5)	319 (70.3)	32 (72.7)	
Adjuvant treatment				0.305
No	228 (46.1)	204 (45.3)	24 (53.3)	
Yes	267 (53.9)	246 (54.7)	21 (46.7)	
Neoadjuvant treatment				0.060
No	432 (86.4)	389 (85.5)	43 (95.6)	
Yes	68 (13.6)	66 (14.5)	2 (4.4)	

BMI: body mass index; CEA: carcinoembryonic antigen.

**Table 2:** Short-term and long-term outcomes after curative resection in patients with and without previous history of hemorrhoid.

Clinical outcomes	Overall population	Previous history of hemorrhoid		*P* value
		No	Yes	
	(*N* = 510)	(*N* = 465)	(*N* = 45)	
Anastomotic complication^1^				0.615
No	443 (86.9)	405 (87.1)	38 (84.4)	
Yes	67 (13.1)	60 (12.9)	7 (15.6)	
Anastomotic leakage				0.349
No	475 (93.1)	431 (92.7)	44 (97.8)	
Yes	35 (6.9)	34 (7.3)	1 (2.2)	
Postoperative fever^2^				0.569
No	355 (69.6)	322 (69.2)	33 (73.3)	
Yes	155 (30.4)	143 (30.8)	12 (26.7)	
Pelvic abscess				1.000
No	490 (96.1)	446 (95.9)	44 (97.8)	
Yes	20 (3.9)	19 (4.1)	1 (2.2)	
Alive status				0.032∗
Alive	401 (78.6)	360 (77.4)	41 (91.1)	
Death	109 (21.4)	105 (22.6)	4 (8.9)	
Total recurrence^3^				0.023∗
No	368 (72.2)	329 (70.8)	39 (86.7)	
Yes	142 (27.8)	136 (29.2)	6 (13.3)	
Local recurrence				0.679
No	442 (89.3)	401 (89.1)	41 (91.1)	
Yes	53 (10.7)	49 (10.9)	4 (8.9)	
Distant metastasis				0.047∗
No	395 (79.8)	354 (78.7)	41 (91.1)	
Yes	100 (20.2)	96 (21.3)	4 (8.9)	

^∗^ Statistically significant *P* value. (1) Anastomotic complication refers to anastomotic leakage, bleeding, or stenosis after operation in this study. (2) Postoperative fever was defined as a body temperature ≥38.5°C within seven days after operation. (3) Total recurrence refers to patients with local recurrence and/or distant metastasis during follow-up after operation.

**Table 3:** Univariate analysis of prognostic factors for DFS and OS.

Prognostic factors	Disease-free survival			Overall survival		
	3-year rate (%)	HR (95% CI)	*P* value	3-year rate (%)	HR (95% CI)	*P* value
Age	—	1.00 (0.99-1.02)	0.185	—	1.03 (1.01-1.04)	<0.001
TNM stage						
III	61.6	2.61 (1.86-3.66)	<0.001	77.5	2.75 (1.85-4.09)	<0.001
I-II	83.2	1		89.8	1	
Differentiation degree						
Low	58.1	1.98 (1.37-2.88)	<0.001	74.4	2.22 (1.47-3.36)	<0.001
Moderate/high	77.4	1		87.2	1	
Lymphovascular invasion						
Positive	52.7	2.41 (1.54-3.77)	<0.001	69.6	2.60 (1.61-4.19)	<0.001
Negative	76.1	1		86.1	1	
Perineural invasion						
Positive	33.8	2.41 (1.54-3.79)	<0.001	74.5	1.84 (1.05-3.24)	0.033
Negative	73.4	1		85.6	1	
Preoperative CEA						
>5 ng/ml	64.7	1.78 (1.25-2.53)	0.001	76.4	1.94 (1.29-2.91)	<0.001
0-5 ng/ml	78.5	1		90.2	1	
Hemorrhoid history						
Yes	86.4	0.39 (0.17-0.88)	0.018	95.3	0.33 (0.12-0.92)	0.034
No	72.8	1		83.5	1	

The Cox survival analysis has been applied to all clinicopathological variables, and only significant results were shown in this table. CEA: carcinoembryonic antigen; HR: hazard ratio; CI: confidential interval.

## Data Availability

The datasets used and analyzed during the current study are available from the corresponding author on reasonable request.

## Additional Points

*What does this paper add to the literature?* This study firstly showed patients could get more access to cancer screening from hemorrhoid in rectal cancer and thus improve survival, which supports performing cancer screening in hemorrhoid and general populations.
